# Testosterone modulation of default mode network and its effects on cognitive function and mood in humans: a systematic review

**DOI:** 10.3389/fnins.2026.1901486

**Published:** 2026-09-14

**Authors:** Daria Malich, Andrii Vasylyshyn

**Affiliations:** 1 Institute of Psychiatry, Psychology and Neuroscience, King's College London, London, United Kingdom; 2 Faculty of Medicine, Ivano-Frankivsk National Medical University, Ivano-Frankivsk, Ukraine

**Keywords:** cognition, default mode network, DMN, mood, testosterone

## Abstract

Testosterone exerts widespread effects on brain structure and function, yet its influence on large-scale neural networks, particularly the default mode network (DMN), remains poorly understood. The role of the DMN in self-referential processing, social cognition, and mood regulation has been demonstrated over the last decade. Neuroimaging studies suggest a link between testosterone and large-scale brain networks, particularly the DMN, in cognitive function and mood domains. A systematic search of the PubMed database was conducted from March to April 2026, restricted to human neuroimaging studies published within the past 15 years, in accordance with PRISMA guidelines. A total of 10 studies were included in this review. Risk of bias was assessed using RoB 2, the Newcastle–Ottawa Scale, the NIH Quality Assessment Tool, and the AXIS tool, selected according to study design. Testosterone-related DMN alterations were observed across key self-referential hubs, including the medial prefrontal cortex, anterior cingulate cortex, precuneus, and angular gyrus. Effects were highly sex-specific, developmental stage-dependent, and sensitive to the form and magnitude of androgen exposure. Testosterone levels were mostly linked to changes in affective reactivity and self-referential processing, including aggression modulation and body self-identification. Overall, testosterone-related modulation of DMN connectivity appears to be highly context dependent, with functional consequences predominantly affecting self-referential and affective domains rather than externally oriented cognition. The high heterogeneity of the included studies, alongside the absence of wider hormonal profiling in many studies, constrains causal inference. Future research should prioritize randomized longitudinal designs with comprehensive hormonal assessment and balanced sex representation to clarify the therapeutic relevance of testosterone-related DMN modulation.

**Systematic review registration:**

Identifier: CRD420261368147.

## 1 Introduction

Testosterone is a sex hormone crucial for maintaining healthy reproductive function among males and females. However, over recent decades, growing evidence has demonstrated the vital role of sex hormones in a wide range of cognitive functions, including episodic memory, attention, executive function, and self-referential thinking (Beauchet, 2006; Frick, 2015; Sherwin, 2003). Moreover, sex hormones, such as estradiol and testosterone, which are present in both males and females at different concentrations, have been shown to directly influence neural architecture across the lifespan (Nguyen et al., 2013; Cai and Li, 2020). Via androgen receptors distributed throughout the nervous system, testosterone modulates synaptic plasticity and may contribute to disorders, including Alzheimer's disease (AD), schizophrenia, and autism spectrum disorder (Kuwahara et al., 2021). A growing body of evidence has shown a link between endogenous testosterone levels and verbal fluency, visuospatial ability, episodic memory, and executive function performance (Yeap and Flicker, 2022; Zhang et al., 2020). In a large-scale study of elderly men, testosterone levels were associated with processing speed, sustained attention, and working memory, suggesting that declining bioavailable testosterone levels are correlated with an increased risk of cognitive impairment and dementia (Giannos et al., 2022). A substantial proportion of testosterone's central effects are mediated through aromatization to estradiol by the enzyme aromatase, which is highly expressed in cortical and limbic regions (Bendis et al., 2024). Therefore, the neural effects attributed to testosterone should be considered within the broader hormonal environment rather than in isolation (Bendis et al., 2024). Supporting this concept, dense-sampling neuroimaging studies have shown that day-to-day fluctuations in estradiol are associated with reorganization of large-scale cortical networks, with particularly pronounced effects in regions overlapping the DMN (Pritschet et al., 2020). Dopaminergic signaling in the mesolimbic and mesocortical pathways is modulated through the effects of testosterone on androgen receptors localized across key dopaminergic circuit areas, such as the nucleus accumbens and ventral tegmental area, which are known for their functions in reward processing, motivation, and executive control (Becker and Hu, 2008). Additionally, testosterone has been reported to contribute to alterations in excitation-inhibition homeostasis in the hippocampus and prefrontal cortex through modulation of GABAergic signaling via GABA-A receptors and glutamatergic transmission via NMDA receptors (Kuwahara et al., 2021; Ooishi et al., 2011). A negative correlation between testosterone levels and serotonin 4 receptor binding, particularly in the hippocampus, was found, indicating the important role of testosterone in serotonergic signaling (Perfalk et al., 2017).

Recent evidence suggests that cognitive and affective processes, such as self-referential thought, executive control, and mood regulation, are not localized to a single structure and instead emerge from the coordinated activity of distributed, whole-brain functional networks (Popp et al., 2024; Baptista et al., 2026). DMN connectivity has been directly linked to individual differences in cognitive functioning across development and aging, supporting network-level connectivity as a meaningful unit of analysis for understanding testosterone-related neurocognitive effects (Baptista et al., 2026). Therefore, examining large-scale brain networks may provide a promising framework for identifying the mechanisms of interaction between testosterone and cognition/mood domains. Several studies have shown that complex large-scale functional network interactions in the brain underlie cognitive functions and behavior (Bullmore and Sporns, 2009; Sporns, 2011). Emerging neuroimaging evidence from a placebo-controlled resting-state fMRI study of 96 healthy men has shown that exogenous testosterone administration resulted in disruption of fronto-subcortical and fronto-parietal connectivity (Votinov et al., 2020). Moreover, sex-specific patterns of amygdala–frontal resting-state connectivity involved in cognitive control were predicted using endogenous testosterone levels (Kogler et al., 2023). In transgender men undergoing testosterone therapy, longitudinal changes in functional brain network connectivity, including shifts in affective and salience circuits following treatment, were reported (Grannis et al., 2021; Maniaci et al., 2024).

The default mode network (DMN) is a functional brain network responsible for self-reflection and mental exploration, serving a critical role in brain development (Azarias et al., 2025). The DMN includes structures such as the medial prefrontal cortex, posterior cingulate cortex, precuneus, and angular gyrus, interconnected into one large-scale brain network (Buckner et al., 2008). A central role of the DMN in social cognition, prospective thinking, and mind-wandering has now been well established (Menon, 2023). Moreover, the DMN has been reported as one network within the triple-network model, which also consists of the salience network and frontoparietal network (Smallwood et al., 2021; Wen et al., 2020). The salience network functions as a dynamic switch that modulates transitions between internally directed processes conducted by the DMN and externally oriented processes conducted by the frontoparietal network (Smallwood et al., 2021; Wen et al., 2020; Goulden et al., 2014). Intracranial recordings provide direct evidence that the anterior insula, the central hub of the salience network, exerts task-dependent influence over both the DMN and frontoparietal network, thereby coordinating the balance between internally and externally oriented processing (Das and Menon, 2024). Several of the cognitive and affective functions, including affective reactivity, threat appraisal, and working memory, are therefore unlikely to depend on the DMN in isolation and instead appear to rely on its dynamic interplay with the salience and frontoparietal networks. DMN connectivity alterations have been documented in Alzheimer's disease, major depressive disorder, and schizophrenia, underscoring its critical function in mood and cognition (Anticevic et al., 2012; Hamilton et al., 2015). DMN activity dysregulation has been identified as a consistent correlate of mood disturbance across clinical and subclinical populations (Kaiser et al., 2015). Pathological over-engagement of the DMN reflects disruption of balance within the triple-network model, resulting in failure of the healthy anticorrelated relationship between the DMN and the frontoparietal network, and correlates directly with depressive symptom severity (Mulders et al., 2015). Another systematic review found a positive effect of antidepressant treatment, showing normalization of DMN hyperconnectivity over time (Dichter et al., 2015). Collectively, these findings suggest that DMN connectivity impairment may be a transdiagnostic neural mechanism underlying mood dysregulation, with potential as a biomarker for therapeutic monitoring (Drysdale et al., 2017). Among the three large-scale brain networks included in the triple-network model, the DMN was selected as the focus of the present review due to its well-established role in self-referential processing and affect regulation (Menon, 2023). In contrast, the frontoparietal network primarily supports externally directed cognitive control and attention-demanding tasks, whereas the salience network is responsible for detecting behaviourally relevant stimuli and coordinating dynamic switching between the DMN and frontoparietal network (Menon et al., 2023; Melero and Sanz-Morales, 2024). Testosterone has also been implicated in several processes associated with DMN function, including self-perception, threat appraisal and aggression regulation (Menon, 2023). Therefore, the overlap between the functional role of the DMN and the reported effects of testosterone provides a rationale for focusing on the DMN as a potential mediator of testosterone-related changes in cognition and mood. The existing evidence provides a strong neurotransmitter-based mechanism of testosterone function in mood and cognition (De Velasco et al., 2026). However, the growing amount of data indicates network-based involvement in cognition (Khetan et al., 2026). Therefore, the present review focuses primarily on the role of testosterone in modulating DMN connectivity and its potential implications for self-referential processing and affective reactivity, while also discussing broader cognitive outcomes where evidence is available.

## 2 Materials and methods

This systematic review adhered to the guidelines of the Preferred Reporting Items for Systematic Reviews and Meta-Analyses (PRISMA) (Page et al., 2021). The review has been registered at PROSPERO (CRD420261368147).

### 2.1 Search strategy

PubMed was used to identify and screen relevant studies for this review. The initial search was conducted from March 2026 through April 2026 and was restricted to human studies published after 2011, within the last 15 years, and written in English. The following keywords were used for the database search: (testosterone OR “exogenous testosterone” OR “testosterone administration” OR “testosterone supplementation” OR “testosterone therapy” OR “testosterone replacement” OR “endogenous testosterone” OR “serum testosterone” OR “circulating testosterone” OR “free testosterone” OR “total testosterone” OR “testosterone levels” OR androgen OR androgens OR “androgen deficiency” OR “androgen deprivation” OR hypogonadism OR hypogonadal OR dihydrotestosterone OR DHT OR “testosterone gel” OR “testosterone propionate” OR “testosterone enanthate”) AND (“default mode network” OR DMN OR “medial frontoparietal network” OR M-FPN OR “resting-state” OR “resting state” OR “functional connectivity” OR “brain connectivity” OR “network connectivity” OR “large-scale network” OR “intrinsic connectivity” OR “brain network” OR fMRI OR “functional MRI” OR “functional magnetic resonance imaging” OR “functional magnetic resonance” OR neuroimaging OR “brain imaging” OR “seed-based” OR “BOLD signal” OR “task-based fMRI” OR “task fMRI”) AND (cognit^*^ OR “cognitive function” OR “cognitive performance” OR “cognitive decline” OR “cognitive impairment” OR “executive function” OR “executive control” OR “working memory” OR “episodic memory” OR “verbal memory” OR “spatial memory” OR “memory consolidation” OR attention OR “inhibitory control” OR “processing speed” OR “cognitive flexibility” OR “decision making” OR “self-referential” OR “self-referential processing” OR “social cognition” OR “theory of mind” OR mood OR “mood disorder” OR “emotional processing” OR “emotion regulation” OR affect OR “affective processing” OR “negative affect” OR “positive affect” OR depression OR “depressive symptoms” OR anxiety OR “well-being” OR wellbeing OR “quality of life” OR “emotional reactivity” OR “stress response” OR rumination OR “mind-wandering”).

Additionally, manual internet and reference list searches of the included studies were conducted to identify studies outside of the database screening. No additional studies identified through manual searching were included in the present systematic review. Two independent reviewers were involved in the two-step screening process, including the initial title and abstract screening and full-text screening. Any discrepancies were resolved through discussion or third-party arbitration.

### 2.2 Inclusion and exclusion criteria

The exclusion criteria were applied to any secondary literature or studies with unreliable methods. Included studies assessed the DMN, measured testosterone, and reported cognitive or mood outcomes. Neuroimaging studies examining structures of the DMN without mentioning DMN in any part of the full text were excluded. The present review targeted studies that provided direct or indirect evidence of relationships between DMN, testosterone, and cognitive/mood domains to provide a wider analysis of this currently underexplored area. Only human studies were included to provide data for potential future clinical trials of testosterone modulation of the DMN. We included studies that assessed DMN activity and/or connectivity, without restricting inclusion to either measurement type.

### 2.3 Data and outcomes extraction

Two independent reviewers were involved in data extraction of the following information: authors, year, journal, title, study design, sample, brain imaging tools used, key findings, reported limitations, testosterone assessment/experimental manipulation methods, DMN-related findings, and Cognitive and Mood Domains. Any discrepancies were resolved through discussion.

### 2.4 Risk of bias assessment

Due to the variety of study designs, four risk of bias assessment tools were used, including the Cochrane risk-of-bias tool for randomized trials (RoB 2), the Newcastle–Ottawa Scale (NOS), the NIH Quality Assessment Tool for Observational Cohort and Cross-Sectional Studies, and the AXIS tool. Randomized clinical trials (RCTs) were assessed using RoB 2. Prospective cohort studies were assessed using the NOS. Observational cohort and mixed observational studies were evaluated using the NIH Quality Assessment Tool, while purely cross-sectional studies were assessed using the AXIS tool. Overall judgments were standardized as low, moderate, or high risk of bias to allow consistent interpretation across the different assessment tools. Each risk-of-bias instrument uses a distinct scoring structure. Raw scores were not pooled numerically across tools. Instead, harmonization was achieved conceptually by mapping each instrument's domain-level or item-level judgments onto a common three-tier framework corresponding to low, moderate, and high overall risk of bias, consistent with recommended practice for evidence syntheses incorporating multiple study designs and their respective risk-of-bias instruments (Kolaski et al., 2023). A study was classified as low risk when it satisfied the equivalent of all or nearly all quality domains on its respective tool, moderate risk when one or more domains raised specific yet non-critical concerns (e.g., inadequate confounding control, unclear blinding, or limited follow-up), and high risk when multiple domains, or a domain considered critical to internal validity (e.g., randomization integrity for RoB 2 or selection bias for the NOS and AXIS), were judged deficient. This qualitative mapping was selected in place of a numerical composite score, as neither RoB 2 nor comparable domain-based tools are intended to generate an overall score by summing individual item responses. The risk of bias assessment was performed by two independent reviewers, and any discrepancies were resolved through discussion or third-party arbitration.

## 3 Results

### 3.1 Study selection

The initial PubMed database search yielded 531 records. Following the abstract and title screening process, 55 studies were extracted for full-text assessment. After full-text screening, 45 studies did not meet the inclusion criteria, resulting in 10 studies included in the present systematic review. A detailed overview of the selection process is presented in Figure 1.

**Figure 1:**
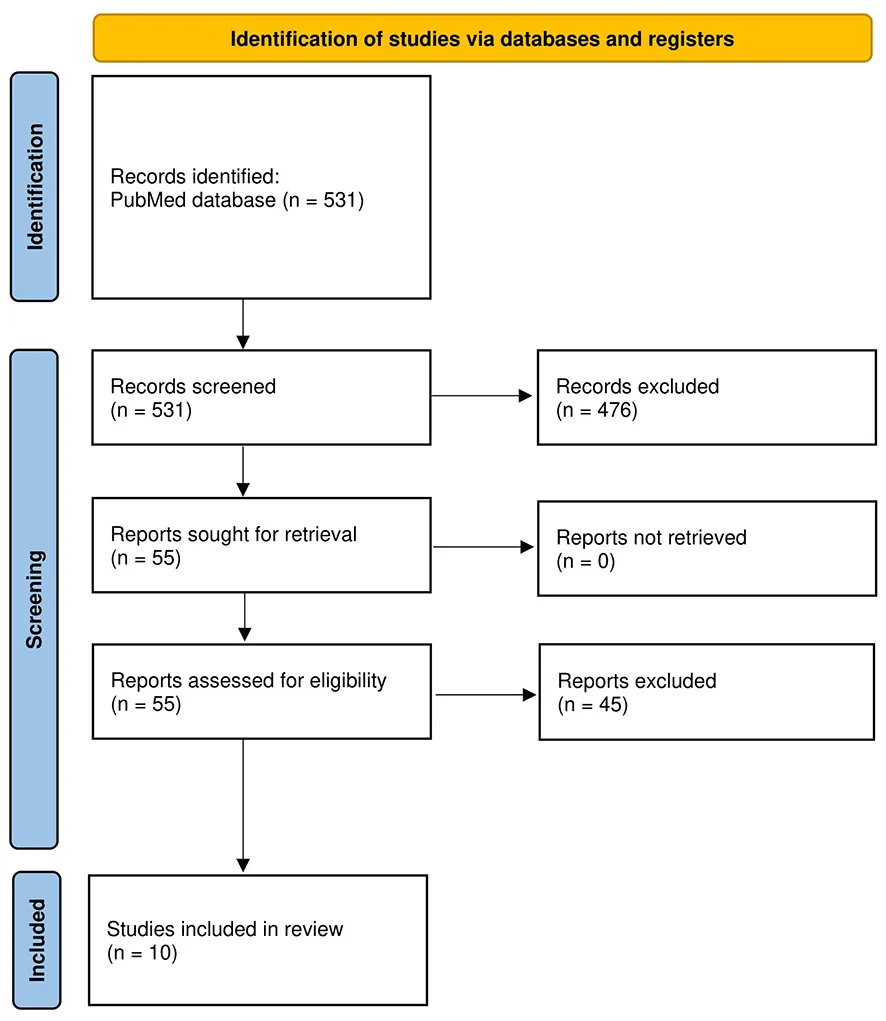
PRISMA 2020 flowchart showing the article screening process.

### 3.2 Study characteristics

The 10 included studies were published between 2014 and 2026 and comprised highly heterogeneous study designs, ranging from a densely sampled single-participant precision-imaging design to a large multimodal cohort of 3,024 adolescent females (Westlye et al., 2016; Khorashad et al., 2021; Lombardo et al., 2020; Pelletier-Baldelli et al., 2024; Wagels et al., 2019; Khetan et al., 2026; Pletzer et al., 2016; Alarcón et al., 2014; Mertens et al., 2025; Grotzinger et al., 2024). Samples included healthy adolescents and adults, transgender individuals undergoing sex-hormone treatment, current and former anabolic-androgenic steroid (AAS) users, women exposed to oral contraceptives, and females across the menstrual cycle. All studies used task-based fMRI or resting-state fMRI as neuroimaging methods, with one study additionally using structural MRI and diffusion MRI/DTI. Cognitive and mood outcomes were measured across working memory, peer evaluation, aggression/provocation, self-referential body perception, overall mood disturbance, and decision-making domains. Testosterone exposure was assessed using a variety of methods, including endogenous serum or salivary levels, fetal testosterone measured from amniotic fluid, exogenous testosterone administration, long-term anabolic-androgenic steroid exposure, cross-sex hormone treatment, and androgenic hormonal contraceptive exposure. Key limitations across most of the studies included small or single-sex samples and the absence of broader hormonal assessment (Table 1).

**Table 1:** Study characteristics.

References	Year	Journal	Title	Study design	Sample	Brain imaging tools used	Key findings	Reported limitations
Westlye et al. (2016)	2016	NeuroImage: Clinical	Brain connectivity aberrations in anabolic-androgenic steroid users	Cross-sectional observational case-control resting-state connectivity study.	151 males engaged in heavy resistance strength training. 50 current AAS users, 16 previous users, and 59 controls in the main comparison.	Resting-state fMRI.	Current AAS users showed reduced functional connectivity, especially between the amygdala and DMN. Reductions were greater with dependency, lifetime exposure, and cycle state.	Observational design prevented causal inference; predispositional confounding was possible; male-only sample limited generalizability.
Khorashad et al. (2021)	2021	Scientific Reports	Cross-sex hormone treatment and own-body perception: behavioral and brain connectivity profiles	Longitudinal pre/post hormone-treatment resting-state connectivity study with cisgender controls and behavioral body-perception testing.	40 transmen, 25 transwomen, 15 cisgender controls.	Resting-state fMRI.	Before treatment, both transgender groups showed weaker DMN connectivity in ACC, mPFC, precuneus, left angular gyrus, and superior parietal cortex versus controls. After treatment, mPFC connectivity and self-identification increased, and by scan 2 the significant group differences disappeared.	The study could not distinguish direct hormone effects from effects mediated by improved body congruence; the control group was small and sex-mixed, increasing the risk of type II error.
Lombardo et al. (2020)	2020	Molecular Psychiatry	Sex-specific impact of prenatal androgens on social brain default mode subsystems	Longitudinal prenatal cohort neuroimaging study linked to an *in vitro* human neural stem cell androgen-response experiment.	64 adolescents (32 males, 32 females; male age mean = 15.42 years, standard deviation = 0.92 years; female age mean = 15.55 years, standard deviation = 1.06 years; age range = 13.22–17.18 years).	Resting-state fMRI.	Increasing fetal testosterone was associated with reduced connectivity between anterior and posterior DMN subsystems in males but not females. Dihydrotestosterone (DHT) also dysregulated genes relevant to DMN circuitry, including MEF2C and other synaptic/E-I balance genes.	Interpretation of sex-specific effects remained limited due to the narrower range of fetal testosterone variability in females, making it unclear whether findings reflect biological differences or exposure levels. Alternative mechanisms, including aromatization to estradiol and RORA-related pathways, were not fully examined.
Pelletier-Baldelli et al. (2024)	2024	Developmental Cognitive Neuroscience	Brain network connectivity during peer evaluation in adolescent females: Associations with age, pubertal hormones, timing, and status	Cross-sectional task-based network connectivity study using multivariate statistical learning with cross-validation.	99 adolescents assigned female at birth, aged 9–15 years, enriched for internalizing symptoms.	Task fMRI.	Salience network connectivity was associated with age, pubertal hormones, and pubertal status; frontoparietal-reward connectivity was associated only with hormones. Cinguloopercular-reward connectivity was related to age and pubertal status, but not hormones or timing.	Model performance was variable; lack of DMN findings may reflect unmeasured environmental factors; medication use may have influenced DMN connectivity; pubertal timing may have been measured imperfectly. Broader hormone assessment and directionality testing were recommended.
Wagels et al. (2019)	2019	Neuropharmacology	Exogenous testosterone and the monoamine-oxidase A polymorphism influence anger, aggression and neural responses to provocation in males	Double-blind placebo-controlled experimental task-fMRI study.	The total of 105 males aged 18–35 were included in the study; 93 healthy males received testosterone or placebo gel.	Task fMRI.	Decision-making after provocation involved core DMN hubs, and testosterone administration increased activation in this network. An interaction between MAOA polymorphism and testosterone was identified in the cuneus, and task-related anger was higher in the short-allele placebo group.	Generalizability remained limited to young men with low–normal testosterone, with potential residual task effects before fMRI. Small and unequal genotype groups reduced statistical power, and lack of molecular analysis limited interpretation of the testosterone–MAOA interaction.
Khetan et al. (2026)	2026	Human Brain Mapping	Pubertal Hormones and the Early Adolescent Female Brain: A Multimodality Brain MRI Study	Cross-sectional multimodal MRI study using ABCD data and elastic-net regression with cross-validation.	3024 adolescent females aged 8.92–13.33 years (mean 10.37).	Structural MRI; diffusion MRI/DTI; resting-state fMRI; task fMRI.	Structural features, white matter microstructure, and resting-state connectivity were important correlates of hormones. Testosterone and DHEA were most strongly linked to parietal and occipital regions. All three hormones were also associated with prefrontal, temporoparietal junction, and insula cortices.	Key limitations were identified, including the use of a female-only sample, lack of control for menstrual cycle phase and intra-individual hormonal variability, variability in sampling time despite known diurnal fluctuations, and the cross-sectional design, thereby limiting causal inference and generalizability.
Pletzer et al. (2016)	2016	Brain Connectivity	Menstrual Cycle and Hormonal Contraceptive-Dependent Changes in Intrinsic Connectivity of Resting-State Brain Networks Correspond to Behavioral Changes Due to Hormonal Status	Mixed design: within-subject menstrual-cycle study plus between-group contraceptive comparison.	18 naturally cycling women scanned at three cycle phases; 32 oral contraceptive (OC) users, including 16 androgenic and 16 antiandrogenic users.	Resting-state fMRI.	Menstrual-cycle and contraceptive-dependent changes were found across several resting-state networks, and these changes paralleled behavioral changes, particularly in the luteal phase. OC-related effects depended on the androgenicity of the progestin component.	Behavioral effects may have been bidirectional depending on baseline dopamine, so opposite-direction effects may have been collapsed within the same group; inconsistencies with prior studies may also reflect study design differences.
Alarcón et al. (2014)	2014	Brain Research	Sex differences in the neural substrates of spatial working memory during adolescence are not mediated by endogenous testosterone	Cross-sectional spatial working memory task-fMRI study with hormone assessment.	74 healthy adolescents aged 10–16 years, including 37 males.	Task fMRI.	There were no sex differences in spatial working memory (SWM) performance, but girls showed stronger deactivation in DMN regions whereas boys showed greater activation in frontal working-memory-related regions. Testosterone did not mediate these sex differences in neural activation.	The sample may have been underpowered to detect hormone effects; testosterone assays may be unreliable; hormones were not collected on scan day; functional connectivity was not directly tested; there was no child/adult comparison; and the cross-sectional design limited developmental inference.
Mertens et al. (2025)	2025	Cerebral Cortex	Sex and age differences on how testosterone relates to brain activity during working memory among adolescents and young adults	Cross-sectional working-memory fMRI study with salivary testosterone assessment.	145 typically developing participants aged 12–25 years, including 74 females and 71 males after exclusions.	Task fMRI.	In females, higher testosterone was associated with stronger ACC deactivation during the most difficult working-memory condition, especially in younger participants. In males, higher testosterone was associated with increased right precentral gyrus activation, without age moderation.	Functional aspects of working memory were assessed, without structural measures. Earlier ages may be needed to detect the onset of testosterone effects and the significant moderation had a small effect size.
Grotzinger et al. (2024)	2024	The Journal of Neuroscience	Diurnal Fluctuations in Steroid Hormones Tied to Variation in Intrinsic Functional Connectivity in a Densely Sampled Male	Case study.	One healthy 26-year-old male studied over 40 sessions across 30 consecutive days.	Resting-state fMRI.	Testosterone, estradiol, and cortisol showed robust diurnal fluctuations that were associated with widespread changes in whole-brain coherence, especially in the Dorsal Attention Network. Compared with a previously studied densely sampled female, hormone-connectivity relationships were stronger in the male participant.	The study could not test causal mechanisms; alertness, respiration, blood flow, melatonin, and sleep-wake preference may also contribute to the results.

### 3.3 Synthesis of DMN findings

#### 3.3.1 Adult men

In current AAS users, a significant reduction in functional connectivity between the amygdala and DMN was observed, showing correlations with dose, dependency, on-cycle status, and an increased testosterone-to-epitestosterone ratio (Westlye et al., 2016). In a double-blind, placebo-controlled RCT, healthy males exposed to exogenous testosterone showed greater DMN activation during decision-making after social provocation compared with the control group, indicating testosterone involvement in DMN activity in the context of reactive aggression (Wagels et al., 2019). In the dense-sampling study of a single male, diurnal testosterone fluctuations showed negative associations with DMN connectivity, although the strongest hormone–brain associations were observed in non-DMN systems (Grotzinger et al., 2024).

#### 3.3.2 Adult women

In female androgenic oral contraceptive (OC) users, alterations across anterior and posterior DMN components, particularly increased mPFC connectivity, were found relative to naturally cycling women (Pletzer et al., 2016). Menstrual phase-related changes in posterior DMN regions, such as the temporal cortex and cuneus/precuneus, were also reported across the natural cycle (Pletzer et al., 2016).

#### 3.3.3 Transgender adults undergoing gender-affirming hormone therapy

In pretreatment transgender participants, weaker connectivity within the DMN was reported, particularly in the ACC, mPFC, precuneus, angular gyrus, and superior parietal cortex, compared with the cisgender control group (Khorashad et al., 2021). Following cross-sex hormone treatment, DMN connectivity increased to levels comparable to those observed in the cisgender control group, showing no longer detectable differences at follow-up (Khorashad et al., 2021).

#### 3.3.4 Adolescents and developmental populations

Endogenous hormone studies in adolescent samples have provided a more complex sex-dependent pattern in DMN changes. Higher fetal testosterone levels predicted reduced connectivity between anterior cortical-midline and posterior DMN subsystems in adolescent males, whereas no such association was present in females (Lombardo et al., 2020). Similar results remained significant when controlling for contemporaneous testosterone levels during adolescence (Lombardo et al., 2020). During a spatial working memory task, adolescent girls showed greater deactivation of DMN regions, including the precuneus and anterior cingulate cortex, whereas boys demonstrated greater frontal cortex recruitment (Alarcón et al., 2014). No significant mediating role of endogenous testosterone in sex differences was found (Alarcón et al., 2014). Similarly, Mertens et al. (2025) reported an association between higher salivary testosterone and stronger ACC deactivation during the working memory task in younger female participants, while higher testosterone in male participants was linked to increased precentral activation. In a large multimodal cohort study of early-adolescent females, testosterone was associated with increased connectivity between the DMN and its posterior subcomponent, the retrosplenial temporal network, alongside decreased connectivity between the DMN and both reward-related and control-related networks (Khetan et al., 2026). Notably, this study assessed testosterone alongside DHEA, and the authors interpreted the two androgens as jointly, rather than independently, related to brain structure, indicating that testosterone-specific attribution of these DMN findings cannot be fully disentangled from co-occurring DHEA exposure (Khetan et al., 2026). In contrast, no effect of testosterone on DMN connectivity was demonstrated in female adolescents during peer evaluation (Pelletier-Baldelli et al., 2024). This study likewise measured pubertal testosterone and DHEA together, and reported that DHEA, rather than testosterone, was the androgen more consistently associated with reward-related network connectivity, again suggesting that testosterone's apparent lack of effect on the DMN in this sample should be interpreted alongside its shared variance with DHEA rather than in isolation (Pelletier-Baldelli et al., 2024). Lastly, the dense-sampling study discussed above under adult men showed negative associations between DMN connectivity and testosterone levels, although the strongest hormone–brain associations were observed in non-DMN systems (Grotzinger et al., 2024) (Table 2).

**Table 2:** Overview of testosterone assessment methods, DMN findings and cognitive/mood outcomes.

References	Testosterone assessment/ experimental manipulation methods	Default mode network (DMN)–related findings	Cognitive and mood domains
Westlye et al. (2016)	High (AAS users, essentially synthetic T). Testosterone to epitestosterone T/E ratio elevated	Reduced connectivity between the amygdala and DMN	Increased aggression/violence reported in AAS users
Khorashad et al. (2021)	Participants received cross-sex hormone treatment involving testosterone administration	At baseline, both transgender groups showed reduced DMN connectivity (including ACC, mPFC, precuneus, and angular gyrus) compared to cisgender controls; following hormone treatment, partial increases in DMN connectivity were observed in transgender participants, with no significant group differences remaining at follow-up	In the DMN, increased connectivity in the precuneus/posterior cingulate cortex was positively associated with improvements in own body perception, such that greater increases in self-perception ratings corresponded to greater increases in DMN connectivity following treatment
Lombardo et al. (2020)	Prenatal (fetal) testosterone (amniotic fluid)	Fetal testosterone (FT) was negatively associated with DMN connectivity in adolescent males, but not in females. This sex-specific relationship remained significant even after controlling for current testosterone levels during adolescence. Current testosterone itself was negatively correlated with DMN connectivity in males only	Dihydrotestosterone (DHT)-dysregulated genes were reported to be enriched for syndromic causes of autism and intellectual disability and to be highly enriched for genes that are spatially expressed in a similar pattern to the cortical midline DMN subsystem
Pelletier-Baldelli et al. (2024)	Pubertal testosterone (saliva)	No significant results involving the DMN was reported. The lack of DMN associations across puberty and age outcomes was unanticipated	The role of the DMN in this process was not directly demonstrated. The reported findings indicate that testosterone was uniquely associated with frontoparietal – reward connectivity, whereas perceived pubertal status and age were associated with cingulo-opercular – reward connectivity. No direct link was found between the DMN and cognitive control
Wagels et al. (2019)	50 mg transdermal testosterone gel (single dose)	An enhanced signal was observed in regions that comprise the core hubs of the DMN, namely the precuneus (extending to the posterior cingulate cortex), posterior angular gyrus, and medial prefrontal cortex. Testosterone increases DMN activity in decision periods. Despite of some evidence for testosterone modulating DMN activity, the biological mechanisms behind this were reported to be unclear	Decision-making following provocation is associated with higher DMN activity
Khetan et al. (2026)	Salivary testosterone	Testosterone was associated with increased connectivity between the DMN and its posterior subcomponent (retrosplenial temporal network), alongside decreased connectivity between the DMN and both reward-related (nucleus accumbens) and control-related (cingulo-opercular) networks	The authors cautiously proposed that androgens (testosterone and DHEA) may be related to cognitive functioning through their associations with brain structure; however, this interpretation was explicitly presented as speculative. No direct link between testosterone-related neural DMN changes and cognitive outcomes was demonstrated
Pletzer et al. (2016)	Users of androgenic oral contraceptives	Users of androgenic but not anti-androgenic OC, showed a significant increase in DMN connectivity compared to naturally cycling women, particularly in the mPFC	Changes in cognitive control were proposed to be influenced by alterations in functional connectivity across brain networks during the luteal phase; however, no direct link was demonstrated in the study
Alarcón et al. (2014)	Saliva testosterone (girls ~3.5 pg/mL, boys ~0.3 pg/mL)	During spatial working memory, sex differences in neural activation were observed, with girls showing reduced recruitment of the dorsolateral prefrontal cortex and greater suppression of DMN regions, while boys demonstrated increased frontal activation and less pronounced DMN deactivation	Differences in spatial working memory were not explained by testosterone levels
Mertens et al. (2025)	Salivary testosterone	In the current study, greater deactivation of DMN regions, including the anterior cingulate cortex (ACC), during a working memory task was observed in female participants ≤ 21 years and was associated with testosterone levels, whereas no such association was found in adult females	Sex differences were observed in the relationship between testosterone levels and brain activation during working memory task performance in adolescents and young adults
Grotzinger et al. (2024)	Serum/Saliva testosterone	Negative associations between testosterone levels and DMN connectivity were observed. These effects were weaker compared to other networks and were more pronounced in males than in females	Total testosterone levels were positively associated with vigor and negatively associated with overall mood disturbance as measured by the Profile of Mood States (POMS), with no other significant relationships between testosterone and mood measures observed after correction for multiple comparisons

### 3.4 Cognitive outcomes

Associations between testosterone-related DMN changes and cognitive outcomes are synthesized below by sex, gender, and developmental/reproductive stage; overall, these associations were less consistent than the network-level alterations themselves (Table 2).

#### 3.4.1 Adult men

No conventional cognitive-performance outcomes tied to DMN changes were reported for adult male samples among the included studies; behavioral outcomes in this group instead centered on affective reactivity and decision-making, addressed in Section 3.5.1 (Westlye et al., 2016; Wagels et al., 2019; Grotzinger et al., 2024).

#### 3.4.2 Adult women

Even though cognitive changes in OC users were proposed to be influenced by large-scale brain networks, no direct link between testosterone-related DMN changes and cognitive function was found (Pletzer et al., 2016).

#### 3.4.3 Transgender adults undergoing gender-affirming hormone therapy

No conventional cognitive-performance outcomes were reported in relation to DMN changes in this population; reported outcomes instead centered on self-referential body perception, addressed in Section 3.5.3 (Khorashad et al., 2021).

#### 3.4.4 Adolescents and developmental populations

No sex differences in spatial working memory accuracy or reaction time were reported, even though divergence in neural recruitment was present, with girls showing greater DMN deactivation and boys greater frontal activation (Alarcón et al., 2014). Likewise, Pelletier-Baldelli et al. (2024) found no correlation between testosterone-related DMN changes and cognitive control. In a study by Mertens et al. (2025), sex- and age-specific neural associations with testosterone during a working memory task were demonstrated. However, no testosterone-related performance advantage was reported (Mertens et al., 2025). In a large early-adolescent female cohort, testosterone-related structural and resting-state features were identified in regions implicated in visuospatial and executive processing, yet task-based emotional n-back activation was not associated with testosterone (Khetan et al., 2026). Moreover, dihydrotestosterone (DHT)-dysregulated genes were enriched for syndromic autism and intellectual disability and showed spatial expression patterns similar to the cortical midline DMN subsystem (Lombardo et al., 2020).

### 3.5 Affective and mood-related outcomes

#### 3.5.1 Adult men

Testosterone administration was associated with increased DMN recruitment during aggressive decision periods, suggesting a possible role of testosterone in anger response modulation (Wagels et al., 2019). In AAS users with chronic supraphysiologic androgen exposure, reduced DMN-amygdala coupling was correlated with aggression, violence, and impulsivity (Westlye et al., 2016). In a single-male study, higher testosterone concentrations were associated with greater vigor and lower overall mood disturbance (Grotzinger et al., 2024).

#### 3.5.2 Adult women

No direct link between testosterone-related DMN changes and affective or mood outcomes was demonstrated in OC users (Pletzer et al., 2016).

#### 3.5.3 Transgender adults undergoing gender-affirming hormone therapy

Improvement in own-body self-identification was positively associated with increased DMN connectivity in the precuneus/posterior cingulate region following hormone treatment, suggesting a link between testosterone-related network reorganization and self-referential body perception (Khorashad et al., 2021).

#### 3.5.4 Adolescents and developmental populations

No affective or mood-related outcomes tied to DMN changes were separately reported for the adolescent/developmental studies beyond the cognitive and network-level findings described above (Alarcón et al., 2014; Mertens et al., 2025; Pelletier-Baldelli et al., 2024; Khetan et al., 2026; Lombardo et al., 2020).

Overall, the included studies suggest that testosterone-related modulation of the DMN may be linked to self-referential processing, affective reactivity, and motivational state, while not being linked to conventional cognitive performance.

### 3.6 Risk of bias

Included studies ranged from low to high risk of bias, with most studies evaluated as being at moderate risk of bias. One study was judged to be at low risk of bias (Lombardo et al., 2020), seven studies were rated as having moderate risk of bias (Westlye et al., 2016; Khorashad et al., 2021; Pelletier-Baldelli et al., 2024; Wagels et al., 2019; Khetan et al., 2026; Mertens et al., 2025; Grotzinger et al., 2024), and two studies were rated as high risk of bias (Alarcón et al., 2014; Pletzer et al., 2016). Across tools, the most recurrent concerns related to limited sample-size justification, cross-sectional designs that constrained causal inference, incomplete adjustment for confounding, uncertainty regarding repeated exposure assessment or adequacy of follow-up, and restricted generalizability due to highly selected, sex-specific, or single-case samples. Detailed risk-of-bias assessment results are provided in the Supplementary material.

## 4 Discussion

The present systematic review synthesized evidence from ten neuroimaging studies exploring the relationships between testosterone and the DMN and the downstream implications for cognitive function and mood. Overall, the evidence yielded mixed findings, indicating the highly context-dependent, sex-specific, and androgen-exposure-sensitive role of testosterone-related DMN changes in cognitive and mood-related outcome domains.

### 4.1 Cognitive outcomes

From a developmental perspective, endogenous testosterone exhibited sex-specific DMN modulation (Lombardo et al., 2020). Higher fetal testosterone levels predicted reduced connectivity between anterior and posterior DMN subsystems in adolescent males, but not in females, an effect that remained significant after controlling for contemporaneous testosterone levels, suggesting a potential contribution of testosterone to neurodevelopmental conditions involving social cognition deficits (Lombardo et al., 2020). However, neither of the two studies evaluating sex differences found a correlation between testosterone-related DMN changes and working memory performance, despite demonstrating different localization patterns of DMN changes between sexes (Alarcón et al., 2014; Mertens et al., 2025).

Connectivity between the DMN and other large-scale networks was also associated with testosterone levels, particularly increased within-DMN and DMN–retrosplenial temporal network connectivity, alongside decreased DMN coupling with reward-related and cognitive control networks (Khetan et al., 2026). These results, in which anterior DMN connectivity strengthens while cross-network integration decreases, may indicate a reconfigurative role of testosterone in the functional balance between internally directed and externally oriented processing streams regulated by the triple-network model (Smallwood et al., 2021). As noted above, this interpretation is tempered by the co-assessment of DHEA in that sample, which limits attribution of these DMN changes to testosterone alone (Khetan et al., 2026).

Still, Pelletier-Baldelli et al. (2024) found no relationship between pubertal testosterone levels and DMN connectivity during peer evaluation in adolescent females, and no association with cognitive control outcomes. Similarly, Pletzer et al. (2016) found no direct connection between testosterone modulation of the DMN and cognitive function. Collectively, these findings suggest that the cognitive consequences of testosterone-related DMN modulation depend on factors including sex, developmental stage, and hormonal context.

### 4.2 Mood and affective outcomes

Most studies reported testosterone-related effects on DMN regions involved in self-referential and affective processing, rather than on externally oriented cognitive performance. Chronic supraphysiologic androgen exposure in AAS users was associated with significantly reduced functional connectivity between the amygdala and the DMN, with the severity of disruption correlating with dose, dependency status, and the testosterone-to-epitestosterone ratio (Westlye et al., 2016). Given the well-established role of the amygdala–DMN axis in anxiety and threat appraisal (Buckner et al., 2008; Zidda et al., 2017), this pattern of reduced coupling may account for the elevated aggression, impulsivity, and violence scores observed in AAS users in that study. Further reinforcing this hypothesis, the role of acute exogenous testosterone administration in aggressive decision-making via increased DMN recruitment was reported in a double-blind, placebo-controlled RCT (Wagels et al., 2019).

In a transgender study, testosterone-related DMN reorganization was demonstrated (Khorashad et al., 2021). Pretreatment individuals demonstrated weaker within-DMN connectivity, particularly in the ACC, mPFC, precuneus, angular gyrus, and superior parietal cortex, relative to cisgender controls (Khorashad et al., 2021). Cross-sex hormone therapy resulted in DMN connectivity changes, making it indistinguishable from the cisgender baseline, while improving own-body self-identification (Khorashad et al., 2021). These findings converge with prior evidence that DMN connectivity is integral to bodily self-consciousness and propose that testosterone may be one hormonal determinant of this process (Menon, 2023).

In a densely sampled male, diurnal testosterone fluctuations were negatively correlated with DMN connectivity and positively associated with vigor and lower mood disturbance scores (Grotzinger et al., 2024). However, other non-DMN networks showed a higher association with mood domains (Grotzinger et al., 2024), suggesting that the affective consequences of testosterone-related DMN modulation, like its cognitive consequences, depend on factors including sex, developmental stage, and hormonal context.

The DMN operates within a broader triple-network framework alongside the salience and frontoparietal networks, whose coordinated interaction underlies complex cognitive and affective function and whose dysregulation is implicated across psychiatric and neurological conditions (Menon et al., 2023). The salience network, anchored in the anterior insula, has been shown to detect behaviorally relevant stimuli and mediate switching between the DMN and the frontoparietal network (Menon et al., 2023), a mechanism supported by causal modeling showing the anterior insula as a directed outflow hub whose inter-network influence predicts task performance (Cai et al., 2021).

Testosterone modulates connectivity beyond the DMN, for instance, exogenous administration reduced frontolimbic and frontoparietal connectivity in men (Votinov et al., 2020), and pubertal testosterone and DHEA were associated with salience-network and frontoparietal–reward connectivity during a socially salient task (Pelletier-Baldelli et al., 2024). Recent findings of increased within-DMN connectivity and decreased DMN coupling with reward and cognitive control networks (Khetan et al., 2026) suggest that testosterone-related DMN alterations may reflect a broader reorganization of salience and frontoparietal network engagement. Future work should model these networks jointly and apply effective connectivity approaches to determine whether testosterone acts primarily on the DMN or on the salience network's capacity to regulate it.

### 4.3 Potential therapeutic implications of testosterone for cognitive performance and mood: benefits, risks, and limitations

#### 4.3.1 Cognitive performance

Testosterone replacement therapy (TRT) is an established intervention for hypogonadism, a condition associated with fatigue, depressed mood, and impaired concentration (Beauchet, 2006; Cai and Li, 2020). Epidemiological evidence suggests an association between low bioavailable testosterone and cognitive impairment, deficits in processing speed, and an increased risk of dementia in older men, raising the question of whether restoration of physiological testosterone levels could mitigate age-related cognitive decline (Giannos et al., 2022; Yeap and Flicker, 2022). Moreover, individuals with amnestic mild cognitive impairment (aMCI) exhibit reduced functional connectivity within DMN regions (Li et al., 2020), including the mPFC, posterior cingulate cortex, and precuneus, which are among the regions most consistently affected by testosterone in the included studies. The neuroprotective role of testosterone has also been demonstrated at the cellular level through multiple converging mechanisms, including suppression of pro-inflammatory cytokines, reduction of oxidative stress via activation of antioxidant enzymes and upregulation of neurotrophic factors essential for neuronal maintenance (Saleki et al., 2022). Additionally, in preclinical models, testosterone has been shown to reduce amyloid-beta (Aβ) production, improve synaptic signaling, and counteract neuronal death (Rosario et al., 2010). Testosterone involvement in adult hippocampal neurogenesis through androgen-dependent pathways has recently been shown to enhance the survival of newly generated dentate gyrus neurons, with indirect involvement of brain-derived neurotrophic factor (BDNF) and glucocorticoid signaling (Spritzer and Roy, 2020). In a recent clinical study, lower testosterone concentrations have been associated with higher amyloid burden and increased AD prevalence, underlining the potential role of testosterone supplementation in the protection of APOE4 carriers from tau pathology accumulation (Shi et al., 2025).

Androgen deprivation therapy (ADT), used to treat prostate cancer, provides consistent evidence of an association between testosterone and cognitive function (Reiss et al., 2023). Several studies have indicated an association between ADT and reductions in verbal and visuospatial memory, executive function, and processing speed, as well as mood changes, fatigue, and an increased risk of dementia (Reiss et al., 2023; Hinojosa-Gonzalez et al., 2024). In a cross-sectional neuroimaging study, ADT-related alterations in brain connectome structure were associated with cognitive decline (Buskbjerg et al., 2021), accompanying the findings of the present systematic review regarding the role of large-scale brain networks in cognitive function and their modulation by testosterone. Findings from both ADT and TRT studies further strengthen the evidence associating testosterone with neuroprotective function and with brain connectivity, particularly within brain networks implicated in cognition and mood.

However, the existing clinical evidence regarding these effects remains heterogeneous. For instance, a meta-analysis of RCTs in cognitively healthy older men found modest improvements in psychomotor speed and executive function in trials that successfully raised total testosterone levels, with no statistically significant effects on verbal memory or visuospatial ability (Tan et al., 2019). In contrast, another RCT reported no improvement in memory or other cognitive functions in the group undergoing testosterone treatment compared with placebo (Resnick et al., 2017). Importantly, one study reported improvements in global cognitive composite scores in the testosterone group relative to placebo, suggesting the crucial role of metabolic and hormonal context in the cognitive benefits of TRT (Gregori et al., 2021). These discrepant findings reflect the heterogeneity of sample characteristics. Therefore, future randomized controlled trials assessing DMN connectivity in the context of testosterone effects should consider measuring the broader hormonal and metabolic profile of patients.

The relationship between testosterone and cognition appears to vary across the human lifespan, highlighting the important role of androgen hormones in early-age neurodevelopment and cognitive maintenance in the elderly population (Wierenga et al., 2018). During puberty, testosterone is associated with neuroarchitectural development in a sex-specific manner, particularly in subcortical and prefrontal structures, which may influence executive function, reward processing, and working memory (Wierenga et al., 2018). Moreover, one systematic review reported sex-specific functional reorganization of higher-order cognitive circuits following pubertal androgen exposure (Vijayakumar et al., 2018).

Population-level data from the UK Biobank, covering 159,411 community-dwelling men followed for a median of seven years, indicated a 43% higher risk of incident all-cause dementia and an 80% greater risk of Alzheimer's disease in men within the lowest testosterone quintile compared to those in the highest quintile (Marriott et al., 2022). Additionally, a meta-analysis of 27 studies reported a significant association between declining total testosterone and elevated all-cause dementia risk (Zhang et al., 2020).

Higher total and free testosterone levels were associated with higher scores in episodic memory and processing speed assessments in older men, while no association between testosterone and cognitive task performance was found in an age-matched women group (Dong et al., 2021). However, this sex specificity is further complicated by genotype. For instance, in older women, lower testosterone was associated with poorer global cognition, processing speed, and verbal memory among APOE-ε4 carriers only (Dratva et al., 2024). A recent longitudinal study demonstrated a more complex relationship between testosterone and cognition in women, with higher testosterone levels linked to poorer visuoconstruction and verbal fluency (Bahr et al., 2025).

These findings suggest a crucial and complex connection between testosterone and cognitive function in both men and women across the lifespan.

#### 4.3.2 Mood

Studies by Khorashad et al. (2021) and Grotzinger et al. (2024) provide evidence that testosterone-related DMN changes may accompany mood improvement. These observations are consistent with broader evidence of DMN hyperconnectivity normalization mechanisms mediated by antidepressants (Dichter et al., 2015) and with DMN dysregulation as a potential mechanism underlying mood disorders (Kaiser et al., 2015). Clinically, testosterone treatment was associated with significant reductions in depressive symptoms in men across a broad age range, manifesting more robustly in hypogonadal populations (Walther et al., 2018).

Despite the large amount of evidence, several significant risks and limitations substantially constrain the therapeutic utility of testosterone-based interventions. For instance, evidence from AAS users demonstrated increased aggression, impulsivity, and violence associated with the effects of supraphysiological testosterone exposure on DMN-amygdala connectivity (Westlye et al., 2016). The divergent DMN responses to testosterone levels imply the need for sex-tailored and individualized approaches in dosing protocols (Alarcón et al., 2014; Mertens et al., 2025; Lombardo et al., 2020). Another TRT risk is testosterone-induced erythrocytosis, which elevates blood viscosity and is associated with an increased risk of major adverse cardiovascular events (MACE) and venous thromboembolic events (VTE) in the first year of TRT, particularly when hematocrit rises substantially (Ory et al., 2022). According to the Endocrine Society guidelines, TRT should be avoided in men with a baseline hematocrit above 50% (Bhasin et al., 2018).

### 4.4 Limitations and future directions

Several limitations of this review should be acknowledged. First, only PubMed was searched, a decision specified *a priori* in the PROSPERO registration. We recognize that multi-database searching is generally recommended to minimize the risk of missing eligible studies. However, several features of the present review reduce this risk. PubMed/MEDLINE indexes the great majority of biomedical and neuroimaging journals relevant to this topic. The search string also used extensive synonym expansion across all three concept domains (testosterone/androgen exposure, DMN/connectivity terminology, and cognitive/mood outcomes), increasing sensitivity within the single database searched and we supplemented this with manual reference-list screening of all included studies, which did not identify additional eligible records. Nonetheless, databases such as Embase, Scopus, Web of Science, and PsycINFO index additional journals not captured by PubMed and their omission remains a limitation that could affect the findings of this review. Second, the search was restricted to studies published in English. This restriction was applied for practical reasons: it allowed both reviewers to directly assess study eligibility, extract data, and appraise risk of bias without relying on translation, which would have introduced additional resource requirements and potential translation-related error into the screening and extraction process. We acknowledge that this restriction may have excluded relevant non-English studies, particularly given that testosterone-DMN research is an active area internationally, and that this introduces a potential source of language bias into the review. We report both limitations explicitly and recommend that future updates of this review incorporate multi-database, multi-language search strategies to verify the stability of the present synthesis. Only ten studies met the inclusion criteria, reflecting the limited and emerging nature of research on testosterone, DMN connectivity, and cognitive or mood outcomes. Secondly, due to the heterogeneity of the included studies (e.g., different testosterone exposures, wide variation in age, sex, and hormonal status among study populations), no meta-analysis was conducted, limiting direct comparability. Additionally, many studies did not assess the broader hormonal environment, including estradiol and other sex hormones that interact with testosterone, thereby reducing confidence in attributing the observed neural effects specifically to testosterone. This is a substantial limitation, since several endocrine variables beyond circulating testosterone itself are known to shape DMN and other large-scale network connectivity and were not consistently measured or controlled for across the included studies. A meaningful proportion of testosterone's central effects are mediated indirectly through aromatization to estradiol, and dense-sampling neuroimaging work has shown that day-to-day fluctuations in estradiol across the menstrual cycle drive reorganization of cortical networks, with especially pronounced effects in DMN hubs that are densely populated with estrogen receptors (Pritschet et al., 2020). Brain aromatase converts testosterone into estradiol locally within DMN-relevant regions such as the hippocampus, amygdala, and cortex, meaning androgen-only assessment may fail to capture the estrogenic pathway through which testosterone influences neural connectivity (Azcoitia et al., 2021). Cortisol is another relevant confound, as experimental cortisol administration alters resting-state connectivity of the DMN and its coupling with the salience network, indicating that unmeasured hypothalamic-pituitary-adrenal axis activity could confound testosterone-DMN associations (Runyan et al., 2023). Similarly, circadian and diurnal variation in steroid hormone secretion, menstrual cycle phase, and DHEA levels were inconsistently reported or controlled for across the included studies, further limiting the ability to isolate testosterone-specific effects on DMN connectivity from these co-varying endocrine influences. Finally, many studies were considered to have a moderate or high risk of bias, mainly due to small sample sizes and imbalanced sex representation.

Future research should focus on longitudinal and randomized studies, balanced participant representation, and comprehensive hormonal profiling that extends beyond testosterone alone to include estradiol, sex hormone-binding globulin (SHBG), cortisol, DHEA, menstrual cycle phase, circadian sampling time, and indices of testosterone aromatization, given the demonstrated influence of these endocrine variables on functional connectivity. Preregistration and standardized reporting practices may also improve reproducibility and help clarify the causal role of testosterone in DMN-related cognitive and mood changes.

## 5 Conclusion

This systematic review characterized the neurobiological effects of testosterone on DMN organization, demonstrating alterations in functional connectivity across key DMN hubs involved in self-referential processing, including the medial prefrontal cortex, anterior cingulate cortex, precuneus, and angular gyrus. However, the directionality and behavioral relevance of these effects varied according to sex, developmental stage, and the magnitude and context of androgen exposure, underscoring the complexity of testosterone-mediated neural modulation.

Across the included studies, testosterone-related DMN alterations were more consistently associated with affective and self-related processes, including mood regulation, aggression, and body self-identification, whereas associations with externally oriented cognitive functions remained limited. These findings position the DMN as a potential neural substrate underlying testosterone-dependent modulation of affective regulation and self-processing, while broader cognitive effects likely involve additional mechanisms beyond DMN connectivity changes.

Despite its potential implications for understanding testosterone-related behavioral and clinical phenotypes, the current evidence remains constrained by methodological heterogeneity, limited endocrine characterization, and the predominance of observational designs. Future longitudinal and randomized investigations integrating comprehensive hormonal profiling with multimodal network analyses will be required to clarify the causal mechanisms underlying testosterone-induced alterations in brain network organization and their relevance for cognitive and mood-related disorders.

## Data Availability

The original contributions presented in the study are included in the article/Supplementary material, further inquiries can be directed to the corresponding author.
